# Gastroesophageal Reflux in Critically Ill Children: A Review

**DOI:** 10.1155/2013/824320

**Published:** 2013-01-31

**Authors:** Maria José Solana García, Jesús López-Herce Cid, César Sánchez Sánchez

**Affiliations:** ^1^Pediatric Intensive Care Department and Gastroenterology Section, Gregorio Marañón University General Hospital, 28009 Madrid, Spain; ^2^Servicio de Cuidados Intensivos Pediátricos, Instituto de Investigación del Hospital Gregorio Marañón, Hospital General Universitario Gregorio Marañón, Dr. Castelo 47, 28009 Madrid, Spain

## Abstract

Gastroesophageal reflux (GER) is very common in children due to immaturity of the antireflux barrier. In critically ill patients there is also a high incidence due to a partial or complete loss of pressure of the lower esophageal sphincter though other factors, such as the use of nasogastric tubes, treatment with adrenergic agonists, bronchodilators, or opiates and mechanical ventilation, can further increase the risk of GER. Vomiting and regurgitation are the most common manifestations in infants and are considered pathological when they have repercussions on the nutritional status. In critically ill children, damage to the esophageal mucosa predisposes to digestive tract hemorrhage and nosocomial pneumonia secondary to repeated microaspiration. GER is mainly alkaline in children, as is also the case in critically ill pediatric patients. pH-metry combined with multichannel intraluminal impedance is therefore the technique of choice for diagnosis. The proton pump inhibitors are the drugs of choice for the treatment of GER because they have a greater effect, longer duration of action, and a good safety profile.

## 1. Introduction

Gastroesophageal reflux (GER) occurs when gastric contents pass through the lower esophageal sphincter (LES) into the esophagus [1]. Under normal conditions, reflux is prevented by correct function of the gastroesophageal junction, also known as the antireflux barrier.

## 2. Incidence

GER is very common in children due to immaturity of the antireflux barrier. Clinical manifestations usually begin at 2 to 3 months of age [2] and are characterized by the regurgitation of milk, mostly in the postprandial period; however, the child's growth and development are not affected [2].

The frequency of GER is higher in infants than in older children and adults, with prevalences of up to 85% [3]. The male-to-female ratio is from 1.6 to 1. The higher prevalence is due to immaturity of the esophagus and stomach in infants and because most of the diet is ingested in liquid form [4].

Other risk groups include children with cerebral palsy, children requiring surgery to correct esophageal atresia, and patients with hiatus hernia [2]. The administration of certain drugs that can relax the LES will also predispose to GER. These drugs include the anticholinergics, calcium-channel blockers, benzodiazepines, and dopamine [5]. Additional risk factors that have been identified in adults are alcohol consumption, smoking, connective tissue diseases (particularly scleroderma) [6], and chronic obstructive pulmonary disease [7].

## 3. Pathophysiology

The antireflux barrier is formed by the lower esophageal sphincter (LES) and the diaphragmatic crural sling, which open during swallowing to permit the passage of the food bolus [8]. Opening of the gastroesophageal junction depends on 3 factors: relaxation of the LES, inhibition of the diaphragmatic crural sling, and shortening of the esophagus [8, 9]. A fourth element, the positive pressure gradient present between the stomach and the gastroesophageal junction, also plays an important role [8].

The muscularis propria of the esophagus is formed of a circular muscle layer that generates pressure waves that transport food bolus and a longitudinal muscle layer that acts to shorten the esophagus. Synchrony between the 2 muscle layers produces effective peristalsis, which has a major influence on the pathophysiology of GER, as it avoids the harmful effects of acid reflux on the mucosa and prevents the appearance of complications such as esophagitis and stenosis.

There are 3 basic mechanisms that can lead to GER:

- transient relaxation of the LES
- a transient increase in abdominal pressure that momentarily exceeds the competence of the sphincter
- low basal LES tone.

The most common cause of GER is transitory relaxation of the LES [10] although there are other factors that can also favor reflux, such as the placement of nasogastric tubes, slow gastric emptying [11, 12], neuronal and/or muscle dysfunction [13], and drug- or hormone-induced dysmotility [2]. 

Transitory episodes of relaxation of the LES can not only occur in children in association with swallowing, but can also develop when the stomach is distended by air or fluid. It would appear that a vagal mechanism (neither cholinergic nor adrenergic) is involved in LES relaxation, and nitric oxide may also be implicated [14].

During the initial weeks of life, it is already possible to detect the basal tone of the LES, which would indicate that GER occurs due to a transitory but repetitive loss of pressure caused by inappropriate relaxation of the LES rather than inadequate basal LES pressure [15].

It is important to take into account the influence of position on GER. A study that investigated the effect of position on GER in 10 healthy preterm infants with a gestational age of 35 to 37 weeks demonstrated that the right lateral position was associated with more episodes of reflux then the left lateral position even though gastric emptying was faster in the right lateral position [15]. Additionally, the short length of the sphincter at this age and the lower efficacy of peristalsis, which leads to poor clearance of the refluxed material, mean that the incidence of reflux is higher.

GER in childhood usually resolves spontaneously between 12 and 18 months of age due to growth of the esophagus, an increase in LES tone, a solid diet, and less time spent in the supine position [16, 17].

## 4. Clinical Manifestations of Gastroesophageal Reflux in Children

In some series, the prevalence of symptoms of GER can be as high as 60% [18].

In adults, the most common symptoms of GER are heartburn and regurgitation; dysphagia, odynophagia, and chest pain are less frequent. Erosive esophagitis is present in 30% of patients with GER; the main cause is excessive and persistent presence of acid in the lower part of the esophagus. The maintenance of a gastric pH above 4 is therefore the most important strategy for controlling this disease.

The most common clinical manifestations in children are the following.

### 4.1. Digestive Tract Manifestations [2]

They are as follows

- vomiting and regurgitation: these are the most common manifestations in infants and are considered pathological when they have repercussions on the infant's nutritional status
- irritability and food rejection
- heartburn, dysphagia, and retrosternal pain, mainly in older children
- erosive esophagitis, Barrett's esophagus, and esophageal stenosis, which are uncommon in children [14, 19].

### 4.2. Respiratory Tract Manifestations [2]

They are as follows

- asthma and chronic cough
- laryngitis and stridor
- aspiration pneumonia
- apnea.

## 5. Diagnosis

It is essential to take an adequate history that details the nature and frequency of vomiting [2].

### 5.1. Imaging Studies with Contrast

These studies are mainly used to exclude obstruction. They are not the methods of choice for the diagnosis of GER as they have a high rate of false positives and negatives [2, 20].

### 5.2. Gamma Scan

Gamma scan studies can detect slowing of gastric emptying and the presence of silent aspiration; however, it is not routinely indicated for the diagnosis of GER in children [2, 20].

### 5.3. Esophagogastroduodenoscopy

Endoscopy can be used to evaluate the state of the esophagus and degree of esophagitis as well as for identification or exclusion of other causes of esophagitis. In addition, the technique is useful for monitoring the clinical course of Barrett's esophagus [2, 20]. Endoscopy is only indicated if there is a suspicion of complications of GER.

### 5.4. pH-Metry

Until recently, monitoring the pH of the esophagus was considered to be the reference technique for the diagnosis of GER in children. This method detects acid reflux [21]. An episode of acid reflux is defined as a fall in the esophageal pH to below 4 for at least 5 seconds [18, 22].

Some authors consider the presence of more than 9 episodes of acid GER per day to be pathological in children [23]. Although the diagnostic criteria for GER in children vary, the most widely used at the present time for the diagnosis of this condition are the ones published by Boix-Ochoa [24] and by Vandeplas [25], which differ only slightly. The Boix-Ochoa scale integrates the mean duration of the episodes of GER, the clearance time, and the total time of GER to produce a score that is considered to be pathological at values over 6.6.

The most significant limitation of pH-metry is its poor capacity to detect episodes of alkaline reflux, which are common in infants and in patients on treatment with proton pump inhibitors. In addition, it cannot determine the characteristics of the refluxate (liquid, mixed, gas), the height reached by the refluxate in the esophagus, or its clearance [18].

### 5.5. Multichannel Intraluminal Impedance

Multichannel intraluminal impedance (MII) is a new technique that, in combination with pH-metry, increases the sensitivity and specificity of the detection of GER as it detects both episodes of acid and of alkaline reflux [18, 26, 27].

MII is based on the insertion of a nasogastric catheter with 6 esophageal electrodes and 1 or 2 gastric electrodes. The device provides a continuous recording of the changes in conductivity that occur in the esophagus due to the passage of food or air or due to GER [26].

Impedance is determined by the resistance to the passage of an electrical current between electrodes situated on the catheter inserted into the esophagus. When the esophagus is empty, the impedance is high at its walls that are in contact [28]. A fall in the impedance of greater than 50% with respect to the baseline indicates the passage of a food bolus or other substances [28].

MII classifies the episodes of GER as acid if the refluxate causes the pH in the esophagus to fall below 4, alkaline if the pH rises above 7, and weakly acid if the esophageal pH falls by at least 1 unit but does not fall below 4 [28, 29].

In addition to detecting the content of the bolus, MII can identify its composition, as the impedance of the esophagus falls with liquid and mixed reflux but rises with gas reflux [26, 30]. Furthermore, MII can detect the direction and location of episodes of reflux independently of their pH.

Infants have a higher frequency of alkaline reflux due, in part, to their milk-based diet [31]. This finding explains why the majority of episodes of GER in infants are not detected by pH-metry but can be detected when this technique is combined with MII [32].

Some studies compared MII with pH-metry in children and found that MII was more sensitive than pH-metry for detecting GER in children [18, 28, 31].

The main limitation of MII is that there are no established reference values for children that enable a diagnosis of reflux to be made using this method, and we therefore have to resort to normalized indices for adults [33, 34].

## 6. Treatment of GER

The objectives of the treatment of GER are to relieve symptoms, cure esophagitis if it is present, and treat or prevent complications [35]. Strategies to achieve these objectives include lifestyle changes, pharmacological therapy, and surgery.

### 6.1. General Measures

Recommendations on dietary and lifestyle changes depend on the age of the patient.

In infants, dietary modifications include thickening the milk and the introduction of solid foods although this measure only appears to reduce the symptoms and does not affect the number of episodes of reflux [19]. Wenzl and cols. [36] showed antireflux formulas to be useful in reducing the number and severity of episodes of GER, particularly in cases of nonacid reflux.

The prone position is associated with a lower incidence of GER [37], but this is not recommended in neonates or infants due to its association with sudden infant death syndrome—the supine position is preferred in this age group [38]. The use of the prone position should only be indicated in highly selected cases [19].

Elevation of the head of the bed to 30° does not appear to be an effective measure. A study performed by Bagucka and cols. [39] in 10 infants with continuous monitoring of the esophageal pH compared the horizontal position with an inclination of 30°. The authors analyzed the mean number of episodes of GER in each position and found a statistically significant difference in favor of the horizontal position. Recommendations in older children and adolescents are the same as those for adults: elimination from the diet of substances that relax the LES, such as caffeine, chocolate, alcohol, tobacco, and spices, and the avoidance of overweight [19].

### 6.2. Pharmacological Treatment

#### 6.2.1. Prokinetics

These agents are useful in patients with moderate symptoms [5].

- Cisapride is a serotoninergic agent that increases acetylcholine release in the gastrointestinal tract. It acts on the LES and stomach to improve contractility and gastric peristalsis. In this way it improves symptoms and reduces esophageal and respiratory complications [40]. However, at the present time cisapride is not indicated because it is associated with prolongation of the QT interval, arrhythmias, and sudden death.
- Domperidone is a dopaminergic receptor antagonist that reduces the duration of postprandial reflux. It is metabolized by cytochrome P450, and its plasma levels can therefore be affected by other substances that act on this enzyme. Its most undesirable adverse effect is the onset of extrapyramidal symptoms.
- Metoclopramide is an antidopaminergic agent with serotoninergic and cholinergic effects. It increases LES tone, improves esophageal peristalsis, and accelerates gastric emptying. The adverse effects of metoclopramide include extrapyramidal symptoms and tardive dyskinesia [19].
- Erythromycin, in addition to being an antibiotic, has a prokinetic effect through the direct activation of the motilin receptors [19]. This drug has been shown to be effective for the treatment of dysmotility in premature infants and for diabetic and postoperative gastroparesis [19]. Erythromycin has been shown to be useful in GER as it increases LES tone both during meals and in the postprandial period.

#### 6.2.2. Inhibitors of Gastric Secretion

*H*_2_ *-Receptor Antagonists*

The H_2_-receptor antagonists bind to the histamine receptors of the parietal cells, producing competitive inhibition. This reduces acid secretion both in the basal situation and after stimulation by food, caffeine, insulin, or pentagastrin. The H_2_ antagonists also indirectly reduce pepsin secretion, and they have a cytoprotective effect on the gastric mucosa, favoring healing [41].

*Proton Pump Inhibitors*

The proton pump inhibitors (PPIs) are the drugs of choice for the treatment of gastroesophageal reflux and gastroduodenal ulcer [42–44]. They act by binding selectively and irreversibly to the proton exchange ATPase (H/K ATPase), forming disulphide bridges with the cystine residues of the  *α*  subunit of the ATPase [45].

The advantages of the PPIs over the H_2_-receptor antagonists include a longer duration of action, more potent inhibition of acid secretion in the basal situation and after parietal cell stimulation, no induction of tolerance, and a better safety profile [46].

Five PPIs are available: omeprazole, lansoprazole, pantoprazole, rabeprazole, and esomeprazole. The differences in their molecular structure give rise to variations in their pharmacokinetic characteristics [47].

The PPIs have been shown to be more effective than the H_2_-receptor antagonists for symptom relief and for the treatment of erosive esophagitis in adults [48] and children [49]; this is due to their greater inhibition of acid secretion and their longer duration of action [46, 50]. These 2 groups of drugs were compared in a meta-analysis, which showed that the rates of symptom relief and of healing after 8 weeks of treatment were 77% and 85%, respectively, with the PPIs and 48% and 52%, respectively, with the H_2_-receptor antagonists. In addition, the symptoms of heartburn disappeared more rapidly in patients treated with PPIs [48].

Few studies have analyzed the efficacy of the H_2_-receptor antagonists and the PPIs in GER in children. One study performed in children with peptic esophagitis found that 70% of patients responded to treatment with ranitidine [51]; of the 30% that did not respond, 87% did respond to omeprazole. In another multicentre study of children with chronic esophagitis, omeprazole treatment resolved the esophagitis in 82% of patients and led to an improvement in symptoms (heartburn, epigastric pain, irritability, dysphagia, odynophagia, cough, wheeze, and vomiting) in 93% of cases [49].

There is only 1 study that has compared the efficacy of the H_2_-receptor antagonists with that of the PPIs for the treatment of GER in children. In 32 patients, the efficacy of high doses of ranitidine (20 mg/kg/d) was equal to that of normal doses of omeprazole (40 mg/d/1.73 m^2^) [52].

#### 6.2.3. New Treatments

*Agents That Reduce LES Relaxation*

Gastric distension stimulates afferent vagal fibers that run from the stomach to the solitary tract nucleus (STN) and dorsal motor nucleus, producing a rapid relaxation of the LES [53]. Glutamate and  *γ*-aminobutyric acid (GABA) are the neurotransmitters of the neurons in the STN, and the inhibition of the afferent system is mediated by the GABA_B_ receptors [54]. Baclofen is a GABA_B_ agonist that has been shown to be useful for the treatment of GER in children [55, 56].

*Competitive ATPase Potassium-Channel Blockers*

Soraprazan (BY359), revaprazan (YH1885), AZD0865, and CS-526 block acid secretion through a competitive and reversible inhibition of the potassium channels of the H^+^/K^+^ ATPase [57]. At the present time there is little experience with these drugs.

*H*_3_ *-Receptor Agonists*

These agents have been shown to be able to inhibit acid secretion in animal models [58] and may become a treatment for GER in the future [57].

*Gastrin Inhibitor Drugs*

Gastrin stimulates acid secretion through 3 mechanisms: direct stimulation of the parietal cell, increased histamine release from the enterochromaffin cells, and somatostatin release [57]. Blockade of its receptors (the cholecystokinin 2 receptors) will therefore reduce acid secretion.

Some of the research in this field is aimed at developing an antigastrin vaccine that will stimulate the production of antibodies that neutralize this hormone [59].

#### 6.2.4. Surgical Treatment

Surgery is indicated in children with severe respiratory problems such as aspiration, apnea, or laryngospasm, in patients with a poor pharmacological response due to motor disturbances of the esophagus that provoke continuous aspirations, children who do not tolerate the medication [60], and GER associated with esophagitis, malnutrition, persistent vomiting, or hiatus hernia [2].

In children with neurological disabilities, the indications for performing an antireflux procedure are the following: presence of episodes of apnea, bradycardia, recurrent pneumonia or apparent life threaetning events, Barrett's esophagus, and the presence of a gastrostomy [2].

There are a number of surgical techniques: the Nissen total fundoplication, the anterior partial fundoplication (Thal fundoplication), and the posterior partial fundoplication (Toupet fundoplication) [2]. Laparoscopic surgery is preferred [61, 62]. Fundoplication relieves symptoms in 57% to 92% of patients with a mortality that varies between 0% and 5% [40]. The most common complication is dysphagia [63] although other postoperative complications can occur, such as dumping syndrome, dysphagia, vagal nerve paralysis, hemorrhage, infection, and adhesions [2].

## 7. Gastroesophageal Reflux in the Critically Ill Pediatric Patient

### 7.1. Incidence and Relevance

GER affects up to 25% to 30% of critically ill adults [64] and the refluxate is typically acid in these patients [64].

There is only 1 study that has analyzed the incidence of GER in critically ill children. Abdel-Gawad et al. [65] studied the incidence of GER using pH-metry combined with MII in 24 children on mechanical ventilation. They detected at least 1 episode of GER in 91.6% of patients. On dividing the patients into 2 groups, they found that 100% of patients that developed mechanical ventilation-related pneumonia presented GER compared with 75% of those that did not develop this type of pneumonia.

In our intensive care unit, using pH-metry combined with MII, we detected at least 1 episode of GER in 83% of critically ill children on mechanical ventilation and 36% satisfied the criteria for GER disease.

### 7.2. Characteristics of the Reflux in the Critically Ill Child

In contrast to adults, the episodes of GER in critically ill children are mainly alkaline [65]. This could be due to prophylactic treatment with omeprazole or H_2_-receptor antagonists for digestive tract hemorrhage or to the presence of duodenogastric reflux [66]; however, these factors are also present in critically ill adult patients. This situation explains why impedance has a greater diagnostic sensitivity than pH-metry for the diagnosis of GER in the critically ill child [65].

The ability to clear the refluxate is greater in critically ill children than in adults. In our experience, esophageal clearance was more rapid in children than in adults (30.7 seconds versus 143 seconds) and the total time that the esophageal pH was below 4 was significantly lower (0.96% versus 39.4%).

### 7.3. Pathophysiology

In critically ill patients, GER mainly occurs due to a reduction or loss of pressure in the LES although it can also be triggered by cough and exertion [64]. Other factors that increase the risk of GER are the supine position, the use of large nasogastric tubes [67], adrenergic agonists, bronchodilators, and opiates [64]; the risk of GER is also increased by mechanical ventilation associated with an inhibition of peristalsis and visceral hypoperfusion secondary to the use of PEEP [60], clinical severity [68], shock, sepsis [64], and cranial trauma [69].

In critically ill children we found no relationship between GER and the dose of inotropes or sedatives or the use of mechanical ventilation. Patients receiving muscle relaxants have less GER than other patients; this may be because of the relaxation of the gastric muscles caused by these drugs.

### 7.4. Diagnosis

pH-metry and, particularly, MII are the methods of choice for the diagnosis of GER in the critically ill patient although there are no comparative data for the 2 techniques in adults.

In our experience, MII is able to detect a larger number of episodes of reflux per patient than pH-metry, probably because most of the episodes of reflux in children are alkaline and cannot therefore be recorded by pH-metry. However, although impedance detects a larger number of episodes of reflux overall, it has a lower ability than pH-metry to detect episodes of acid reflux. These results are very similar to those found in studies performed in noncritical children with GER, in whom approximately 70% of the episodes detected by pH-metry alone were not associated with retrograde movement [70].

### 7.5. Complications of GER in the Critically Ill Patient

In adults, GER increases the risk of digestive tract hemorrhage due to damage to the esophageal mucosa and increases the incidence of pulmonary microaspirations and nosocomial pneumonia; these alterations will affect morbidity and mortality, length of hospital stay, and economic cost [64, 71].

Abdel-Gawad et al. [65] found that mortality was higher among children with acid and mixed reflux than amongst those with alkaline reflux. Also, the duration of acid reflux, the number of episodes of acid reflux, the number of episodes of prolonged acid reflux (>5 minutes), the duration of the longest episode of acid reflux, and the acid-reflux index were higher in patients that died than in the survivors. In contrast, the parameters of alkaline reflux did not differ between children that died and survivors. These differences were not detected in our study, but the mortality was much lower (5.5%) than in the study by Abdel-Gawad et al. (58.3%) [65].

### 7.6. GER Prophylaxis and Treatment in the Critically Ill Patient

Elevation of the head of the bed to 45° and the use of small-diameter nasogastric tubes are measures that can reduce the incidence of GER in critically ill patients [23]. The use of transpyloric tubes also helps to reduce GER because there is a smaller gastric residue and thus less gastric distension [72, 73].

The administration of prokinetic drugs can be useful to improve gastric hypomotility. Of these, erythromycin and metoclopramide are the most widely used in critically ill patients [73, 74]. The H_2_-receptor antagonists and proton pump inhibitors are useful drugs for the treatment of GER as they inhibit gastric acid secretion, increase the pressure of the LES, and improve esophageal acid clearance [17]. Inhibitors of acid secretion, such as omeprazole, do not reduce the number of episodes of GER, but cause the episodes to be nonacidic and, therefore, less harmful to the esophageal mucosa [75].

## 8. Conclusions

GER is common in healthy infants and in critically ill children. pH-metry combined with MII is the technique of choice for the diagnosis of GER in children, including those with critical illness, because GER is typically alkaline in these patients. The proton pump inhibitors are the treatment of choice for the prophylaxis and treatment of GER. 
